# Novel diagnostic tools for identifying cognitive impairment using olfactory-stimulated functional near-infrared spectroscopy: patient-level, single-group, diagnostic trial

**DOI:** 10.1186/s13195-022-00978-w

**Published:** 2022-03-08

**Authors:** Jaewon Kim, Dong Keon Yon, Kyu Yeong Choi, Jang Jae Lee, Namwoo Kim, Kun Ho Lee, Jae Gwan Kim

**Affiliations:** 1 Department of Biomedical Science and Engineering, Gwangju Institute of Science and Technology, Gwangju, Republic of Korea; 2 Medical Science Research Institute, Kyung Hee University College of Medicine, Seoul, Republic of Korea; 3 Department of Data Science, Sejong University College of Software Convergence, Seoul, Republic of Korea; 4 Gwangju Alzheimer’s & Related Dementia Cohort Research Center, Chosun University, Gwangju, Republic of Korea; 5 Kolab Inc., Gwangju, Republic of Korea; 6 Department of Neuropsychiatry, Seoul National University Hospital, Seoul, Republic of Korea; 7 Gwangju Alzheimer’s & Related Dementia Cohort Research Center, Department of Biomedical Science, Chosun University, Gwangju, 61452 Republic of Korea; 8 Korea Brain Research Institute, Daegu, Republic of Korea

**Keywords:** Cognitive impairment, Alzheimer’s disease, fNIRS, Mild cognitive impairment

## Abstract

**Introduction:**

Basic studies suggest that olfactory dysfunction and functional near-infrared spectroscopy (fNIRS) can be used as tools for the diagnosis of mild cognitive impairment (MCI); however, real-world evidence is lacking. We investigated the potential diagnostic efficacy of olfactory-stimulated fNIRS for early detection of MCI and/or Alzheimer disease (AD).

**Methods:**

We conducted a patient-level, single-group, diagnostic interventional trial involving elderly volunteers (age >60 years) suspected of declining cognitive function. Patients received open-label olfactory-stimulated fNIRS for measurement of oxygenation difference in the orbitofrontal cortex. All participants underwent amyloid PET, MRI, Mini-Mental State Examination (MMSE), and Seoul Neuropsychological Screening Battery (SNSB).

**Results:**

Of 97 subjects, 28 (28.9%) were cognitively normal, 32 (33.0%) had preclinical AD, 21 (21.6%) had MCI, and 16 (16.5%) had AD. Olfactory-stimulated oxygenation differences in the orbitofrontal cortex were associated with cognitive impairment; the association was more pronounced with cognitive severity. Olfactory-stimulated oxygenation difference was associated with MMSE (adjusted *β* [*aβ*] 1.001; 95% *CI* 0.540−1.463), SNSB language and related function (*aβ*, 1.218; 95% *CI*, 0.020−2.417), SNSB memory (*aβ*, 1.963; 95% *CI*, 0.841−3.084), SNSB frontal/executive function (*aβ*, 1.715; 95% *CI*, 0.401−3.029) scores, standard uptake value ratio from amyloid PET (*aβ*, −10.083; 95% *CI*, −19.063 to −1.103), and hippocampal volume from MRI (*aβ*, 0.002; 95% *CI*, 0.001−0.004). Olfactory-stimulated oxygenation difference in the orbitofrontal cortex was superior in diagnosing MCI and AD (AUC, 0.909; 95% *CI*, 0.848−0.971), compared to amyloid PET (AUC, 0.793; 95% *CI*, 0.694−0.893) or MRI (AUC, 0.758; 95% *CI*, 0.644−0.871).

**Discussion:**

Our trial showed that olfactory-stimulated oxygenation differences in the orbitofrontal cortex detected by fNIRS were associated with cognitive impairment and cognitive-related objectives. This novel approach may be a potential diagnostic tool for patients with MCI and/or AD.

**Trial registration:**

CRIS number, KCT0006197.

**Supplementary Information:**

The online version contains supplementary material available at 10.1186/s13195-022-00978-w.

## Background

Alzheimer’s disease (AD) is the most common type of dementia worldwide [1, 2]. Because of the absence of a definitive treatment method, the current alternative is to diagnose early, detect degenerative changes before they become severe, and delay cognitive decline as much as possible. A decrease in olfactory function before the onset of dementia has been shown in previous studies [3–5]. The olfactory function that is degraded in dementia is not the function of smell, but that of distinguishing odors, and the cause of this degradation is the formation of plaques and entanglements in the olfactory bulb and the inner olfactory cortex, which is involved in memory formation [3–5]. In animal and human autopsy studies, these plaques and tangles have been shown to occur earlier in the posterior nerves than in the cerebral cortex [6]. Additionally, a previous animal study conducted on Tg2576 mice demonstrated a decrease in dopaminergic neurons in the olfactory bulb resulting in overproduction of beta-amyloid precursors, which may lead to a decrease in olfactory discrimination function; the accumulation of beta-amyloid in the olfactory bulb was confirmed upon autopsy of the mice [7, 8]. Therefore, if the olfactory function can be quantitatively measured, it can be detected at the stage of mild cognitive impairment (MCI).

Functional near-infrared spectroscopy (fNIRS) has the unique property of passing light through organ tissues and is subsequently absorbed by hemoglobin in the cerebral cortex, which enables real-time monitoring of hemodynamic changes in the cerebral cortex [9]. In particular, the difference in absorbance spectra between oxy-hemoglobin and deoxy-hemoglobin in the cortical regions can reflect continuous hemodynamic changes, which is useful as a marker of cerebral activity [9]. fNIRS has been used to perform functional activation in the field of neuropsychiatric disorders such as schizophrenia, developmental disorders, affective disorders, and dementia [9, 10].

In this context, fNIRS has multiple benefits over functional magnetic resonance imaging (fMRI), positron emission tomography (PET), and questionnaires for early detection of MCI and/or AD [11]. Previous researchers have suggested that the fNIRS approach stimulated by dual-task walking, N-back task, verbal fluency task, and memory and visuospatial test can be useful for the diagnosis of MCI and dementia [12–17] but these methods can increase the burden of medical providers and patients. However, the olfactory-stimulated fNIRS approach is a novel diagnostic method in which seven photodiodes are attached to the forehead of the patient during the experiment for only 1 min for each cycle (total 3 min for three cycles). The convenience of medical providers, short examination times, and non-invasive nature of the test can be useful to patients and medical providers. This novel diagnostic method is non-invasive, highly portable, has low cost and radiation, requires short examination time, and has fewer constraints on elderly patients who have difficulty sitting down for a long time or filling out questionnaires for examination. These benefits of novel fNIRS techniques provide a potential non-invasive and non-expensive alternative diagnostic methodology or therapeutic monitoring to fMRI, PET, and questionnaires for real-world clinical settings. However, the diagnostic study of fNIRS was limited by the relatively small study population, low-evidence study design, and non-standardization, such as stimulation types and interpretation methods.

Here, we hypothesized that olfactory-stimulated fNIRS is a novel diagnostic tool for the early detection of MCI and/or AD compared with conventional imaging studies. Through a patient-level, single-group, diagnostic intervention trial, we investigated the potential diagnostic efficacy of olfactory-stimulated fNIRS for early detection of MCI and/or AD and provided a standardized diagnostic protocol for olfactory-stimulated fNIRS. Furthermore, we aimed to clarify the diagnostic superiority of fNIRS in patients with MCI and/or AD in a real-world clinical setting.

## Material and methods

### Study design

This study was designed as a prospective, patient-level, single-group, diagnostic accuracy study conducted in elderly volunteers (age >60 years) suspected of declining cognitive function between March 02 and August 30, 2021. Elderly volunteers (age >60 years) suspected of declining cognitive function were defined as those who had been recommended a test for cognitive function by physicians or related medical practitioners but had not yet undergone the test. The trial recruited those from a local community in Gwangju Metropolitan City, South Korea. Candidates were excluded if they had any severe life-threatening disease such as any malignancy or severe head trauma, a physical nasal obstruction with an inability to smell, or an alcohol or drug abuse problem, mental illness with psychosis, severe traumatic brain injury, major depressive disorder, or any other medical or psychological condition that, in the opinion of the investigator, may interfere with assessment of the main result or lead to non-cooperation while answering the questionnaire. Written informed consent was obtained from each participant and legal guardian at the time of enrollment. The study protocol was approved by the Institutional Review Board of the Gwangju Institute of Science and Technology (20210115-HR-58-01-02). The trial was registered with the Clinical Research Information Service of the Republic of Korea (CRIS number: KCT0006197). Our study adhered to the tenets of the Declaration of Helsinki.

### Participants

We recruited 97 elderly volunteers (age > 60 years) with a suspected decline in cognitive function. We assessed all participants by medical interviews with a detailed questionnaire to obtain baseline data on age, gender, education status, occupation, household income, smoking status, and the Charlson comorbidity index score. Cognitive function tests were performed using the Mini-Mental State Examination (MMSE), the Seoul Neuropsychological Screening Battery (SNSB) [18], and the Korean Instrumental Activities of Daily Living (K-IADL) [19], which are commonly used in cognitive function tests in South Korea. Cognitive impairment was defined as a *z*-score (normalized for age and education level) less than −1.0 on at least two of the SNSB tests, which assessed attention, language and related function, visuospatial function, memory, and frontal/executive function (Jak/Bondi comprehensive criteria) [18, 20]. Impairments of daily functioning were defined as a K-IADL score of less than 0.40 [19]. Body mass index was measured, and the apolipoprotein E (*APOE*) genotype was tested from peripheral blood samples, with genotyping performed by extracting two SNP genomes from each individual. All participants underwent three-dimensional brain imaging (MPRAGE; *TR*, 2300 ms; *TE*, 2.143 ms; *TI*, 900 ms; *FA*, 9°; *FoV*, 256 × 256; matrix, 320 × 320; slice thickness, 0.8 mm) using a 3.0 T magnetic resonance (MR) scanner (MAGNETOM Skyra, Siemens Healthineers, Germany). The hippocampal volume was measured from each brain’s MR image using the standard recon-all processing pipeline (FreeSurfer Version 5.3.0, Martinos Center for Biomedical Imaging, USA). In addition, ^18^F-Florbetaben PET amyloid imaging (Discovery STE PET-CT scanner, GE Medical Systems, USA) was performed for all participants. The standard uptake value ratio (SUVR) was calculated and normalized by the cortical amyloid burden from six predefined cortical regions, including the anterior and posterior cingulate, frontal and lateral parietal, and lateral temporal regions with reference to the whole cerebellum [21, 22]. We used an Alzheimer’s Disease Neuroimaging Initiative guideline SUVR cutoff of 1.1 to consider amyloid positivity [23].

The diagnostic criteria for MCI and Alzheimer’s dementia for each group were based on the 2011 National Institute on Aging-Alzheimer’s Association recommendations [24]. Although the criteria for patients with mild cognitive impairment met the core clinical criteria of the NIA-AA, patients were not tested for tau protein. Therefore, the subjects were classified as mild cognitive impairment with moderate probability based on Alzheimer’s mild cognitive impairment criteria for research. Dementia patients were classified according to the NIA-AA diagnostic guideline of probable Alzheimer’s dementia. Participants were classified into four groups: 55 (56.7%) cognitively normal (CN) participants, 26 (26.8%) MCI patients, and 16 (16.5%) AD patients.

### Diagnostic procedure

To measure cortical activation, an fNIRS device was used while detecting olfactory stimulation (N.CER Co., Gwangju, South Korea). A 7-channel NIRS system (two-wavelength LED [Fedy Tech, Shenzhen Fedy Technology Co., Ltd., China] and seven photodiodes [SFH 2201, OSRAM, Germany]) were used for all measurements; the light emitted can penetrate biological tissue and be absorbed by hemoglobin, which has different wavelengths (695 nm ± 20 nm and 830 nm ± 20 nm) when oxygenated and deoxygenated. Therefore, fNIRS can be used to identify cortical changes in oxygenated and deoxygenated hemoglobin [25]. In this study, our probeset was positioned at approximately FP1 and FP2, according to the international 10-20 system [26]. In this location, our probeset was placed on both the eyebrows, and LEDs and photodiodes were placed 1 cm above the forehead to remove the skin signal.

All participants performed olfactory-stimulated fNIRS in two different phases. The rest phase was performed for 40 s, and the olfactory stimulation phase was performed for 20 s. There were two types of olfactory stimulation with sniff stick pens (unscented and peppermint-scented; Burghart Screening 12 Test, MediSense, Netherlands) [27]. We conducted each test three times (total time of olfactory stimulation fNIRS: 3 min).

### Sample size calculation

Since there has been no study on the direct relationship between olfactory-stimulated fNIRS and cognitive impairment, we calculated the sample size based on a previous similar study on the association between verbal fluency task-stimulated fNIRS and cognitive impairment [16]. Originally, we calculated that for two groups (CN versus MCI) to have a 75% power to show a 40% oxygenation difference at a 5% significance level, we would need to enroll at least 15 participants in each group. Finally, considering the difficulty in recruiting patients with AD, we included 55 participants with CN, 25 with MCI, and 16 with AD.

### Statistical analysis

Data was generated using Python, and in order to remove the noise generated by movement, the ± 1 mmol × mm/l channels that occurred simultaneously in oxygenated and deoxygenated hemoglobin were manually interpolated. For preprocessing of the concentration change of oxygenated and deoxygenated hemoglobin, high-frequency artifacts were excluded by applying a moving window of 3 s to the first raw data electrical signal. Then, to rule out a drift in fNIRS, we obtained a baseline value during the rest phase for 40 s. In order to remove the system effect for each channel, the filter was processed below 0.4 Hz and then wavelet transform was performed. In addition, the data were scaled down by measuring the skin signal coming from a nearby channel and then removed from the original signal (C-NIRS algorithm). Then, the average change in values between the stimulation and rest phases for each participant was calculated and set as representative values.

Epidemiologic data are presented as mean and standard deviation (SD) or median and interquartile range. All statistical analyses were performed using SPSS (version 25.0; IBM Corp, Armonk, NY, USA) and R software version 3.1.1 (R Foundation, Vienna, Austria). An analysis of covariance was performed to assess between-group differences in oxygenation differences in the orbitofrontal cortex (CN versus MCI versus AD), unadjusted in the crude model, adjusted for age and sex in model 1, and adjusted for age, sex, education (continuous), household income (low, middle, and high), smoking (never or ex-smoker and current smoker), and Charlson comorbidity index (0, 1, and ≥ 2) in model 2 [28, 29]. We performed linear regression models between stimulated oxygenation difference in the orbitofrontal cortex (continuous) and cognitive impairment-related outcomes (MMSE, SNSB, SUVR, and hippocampal volume; continuous) in model 2. Finally, we used the C-statistic for the prediction model in the diagnosis of AD or MCI (dichotomized value), which was presented as the mean area under the receiver operator curve (AUC) value with 95% confidence intervals (CIs). To test the reliability of our main findings, we analyzed the differential conditions using alternative MCI definitions (Jak/Bondi typical criteria), such as a *z*-score less than −1.5 on at least one of the SNSB tests and using another olfactory stimulation (leather-scented). Two-sided *P-*values < .05 were considered statistically significant.

## Results

### Baseline characteristics

A total of 103 elderly volunteers (age > 60 years) with a suspected decline in cognitive function were screened, and 97 of them met the eligibility criteria. Candidates were excluded if they had any severe or life-threatening disease such as any malignancy or severe head trauma (excluded *n* = 0), a physical nasal obstruction with an inability to smell (excluded *n* = 1), or a serious mental problem that led to non-cooperation while answering the questionnaire (excluded *n* = 5). For the overall trial, a total of 97 participants were recruited, of whom 55 (56.7%) were CN (median age 74.0 years; female 50.9%), 26 (26.7%) were MCI patients (median age 74.0 years; female 50.0%), and 16 (16.5%) were AD patients (median age 76.5 years; female 43.8%) (Table 1).

**Table 1:** Baseline characteristics of participants at enrollment (*n* = 97)

Variables	CN	MCI^a^	AD
Number (%)	55 (56.7)	26 (26.8)	16 (16.5)
Age, years, median (IQR)	74.0 (70.0–79.0)	74.0 (68.0–78.5)	76.5 (74.0–82.0)
Body mass index, kg/m^2^, *n* (%)			
<25 (normal)	35 (63.6)	18 (69.2)	12 (75.0)
≥25 (overweight or obese)	20 (36.4)	8 (30.8)	4 (12.5)
Sex, female (%)	28 (50.9)	13 (50.0)	7 (43.8)
Education, years, median (IQR)	9.0 (6.0–14.0)	12.0 (6.0–12.25)	11.0 (8.0–14.8)
Occupation, *n* (%)			
White collar/professional	20 (36.4)	7 (26.9)	4 (25.0)
Blue collar	27 (49.1)	16 (61.5)	9 (56.3)
Household/student/unemployed	8 (14.5)	3 (11.5)	3 (18.8)
Household income, *n* (%)			
Low (1–29 percentile)	9 (16.4)	7 (26.9)	6 (37.5)
Middle (30–69 percentile)	24 (43.6)	12 (46.2)	5 (31.3)
High (70–100 percentile)	22 (40.0)	7 (26.9)	5 (31.3)
Smoking, *n* (%)			
Never or ex-smoker	52 (94.5)	26 (100.0)	14 (87.5)
Current smoker	3 (5.5)	0 (0.0)	2 (12.5)
Charlson comorbidity index, *n* (%)			
0	20 (36.4)	9 (34.6)	6 (37.5)
1	24 (43.6)	11 (42.3)	5 (31.3)
≥2	11 (20.0)	6 (23.1)	5 (31.3)
APOE4 carrier, *n* (%)	14 (21.2)	18 (85.7)	11 (68.8)
Mini-Mental State Examination, median (range)	28.0 (24.0–30.0)	26.0 (22.0–30.0)	20.0 (10.0–24.0)
Cognitive measures (composite *z*-score), mean (SD)			
SNSB attention	−0.07 (0.92)	−0.52 (0.79)	−0.61 (0.94)
SNSB language and related function	0.51 (0.65)	0.02 (1.22)	−2.18 (3.22)
SNSB visuospatial function	1.01 (0.68)	0.17 (1.69)	−2.45 (5.00)
SNSB memory	0.73 (1.08)	−0.64 (1.46)	−2.73 (1.30)
SNSB frontal/executive function	0.55 (0.80)	−0.47 (1.06)	−2.38 (1.53)
Amyloid PET (standard uptake value ratio), mean (SD)	1.13 (0.16)	1.27 (0.26)	1.39 (0.20)
Hippocampal volume, cm^3^, mean (SD)	7.68 (0.99)	7.01 (1.40)	6.04 (1.27)

*Abbreviations*: *AD* Alzheimer disease, *CN* cognitively normal, *IQR* interquartile range, *MCI* mild cognitive impairment, *SD* standard deviation, *SNSB* Seoul Neuropsychological Screening Battery

^a^The diagnostic criteria for MCI were based on the Jak/Bondi comprehensive criteria

### Primary outcome

**Fig. 1:**
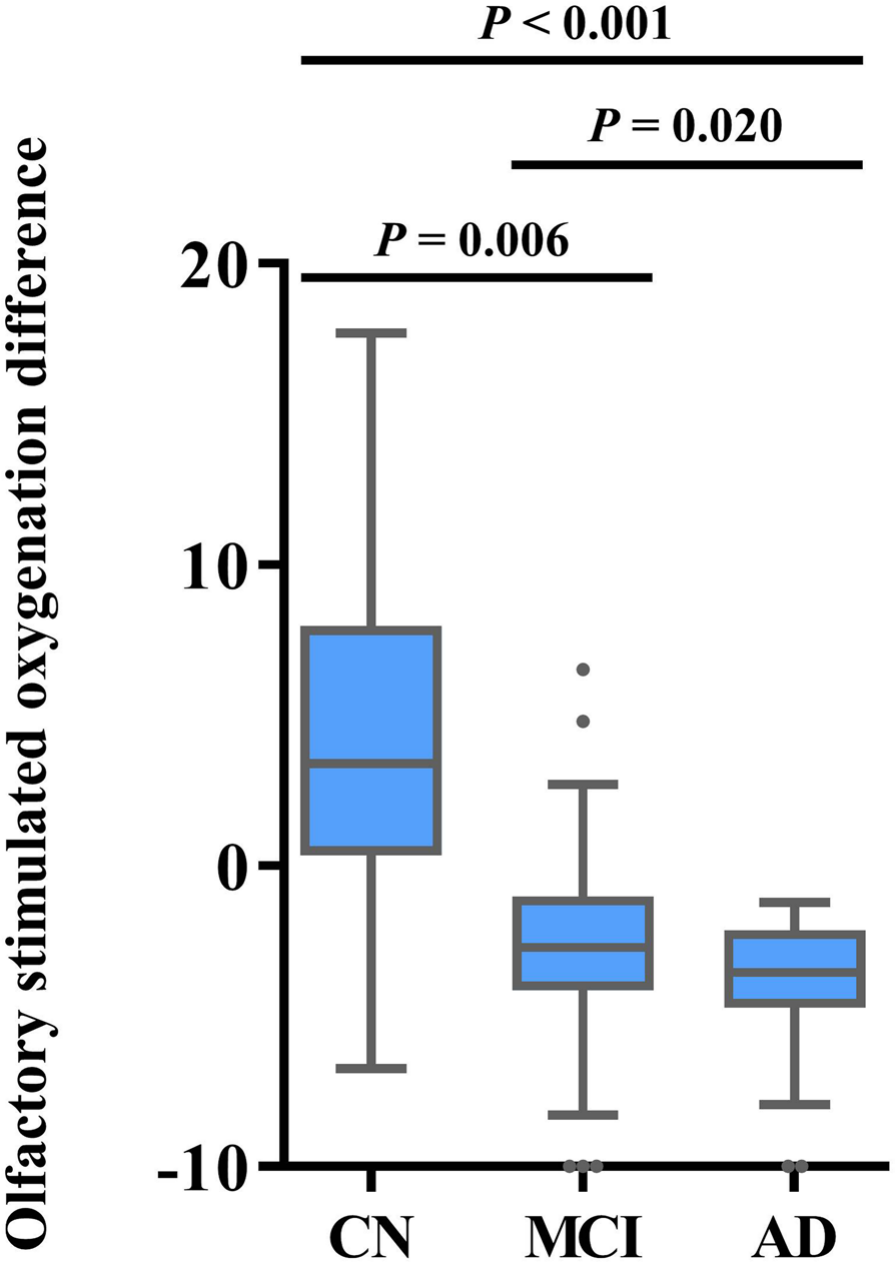
Olfactory-stimulated oxygenation in the orbitofrontal cortex in patients with CN, MCI, and AD. The top and bottom of each box indicate the interquartile range; the I bars represent 1.5 times the interquartile range; the horizontal line inside each box represents the median; the circles represent outliers. These values were analyzed using the Wilcoxon rank sum test

Compared with CN participants (Table 2 and Fig. 1), olfactory-stimulated oxygenation in the orbitofrontal cortex was decreased in patients with MCI (model 2; adjusted mean difference, 10.81; 95% *CI*, 5.27–16.36) and AD (model 2; adjusted mean difference 12.54; 95% *CI* 6.73–18.35). However, no differences were seen in the non-stimulated oxygenation levels in the orbitofrontal cortex in any of the groups.

**Table 2:** Association between stimulated oxygenation difference in the orbitofrontal cortex and cognitive impairment (primary endpoint)

Oxygenation difference in the orbitofrontal cortex	Model	CN	MCI^c^	AD	*P* trend
Olfactory stimulation	Mean (95% *CI*)	5.94 (3.13 to 8.74)	−0.22 (−2.48 to 2.04)	−3.96 (−5.69 to −2.23)	
	Adjusted mean difference (model 1^a^)	1.00 (reference)	**6.12 (2.12 to 10.13)**	**9.93 (5.10 to 14.76)**	**<0.001**
	Adjusted mean difference (model 2^b^)	1.00 (reference)	**6.83 (3.15 to 10.51)**	**9.92 (5.72 to 14.13)**	**<0.001**
None	Mean (95% *CI*)	1.63 (−1.67 to 4.93)	1.92 (−2.46 to 6.30)	0.93 (−1.71 to 3.57)	
	Adjusted mean difference (model 1^a^)	1.00 (reference)	−0.47 (−5.57 to 4.63)	1.32 (−4.83 to 7.46)	**0.872**
	Adjusted mean difference (model 2^b^)	1.00 (reference)	−0.39 (−7.05 to 6.26)	0.51 (−7.11 to 8.12)	**0.870**

Numbers in bold indicate statistically significant associations (*P* < 0.05)

*Abbreviations*: *AD* Alzheimer’s disease, *CN* cognitively normal, *MCI* mild cognitive impairment

^a^Model 1 was adjusted for age and sex

^b^Model 2 was adjusted for age, sex, education (continuous), household income (low, middle, and high), smoking (never or ex-smoker and current smoker), and Charlson comorbidity index (0, 1, and ≥2)

^c^The diagnostic criteria for MCI were based on the Jak/Bondi comprehensive criteria

### Secondary outcome

Among the entire study population (Table 3), the olfactory-stimulated oxygenation difference in the orbitofrontal cortex was associated with MMSE scores (*aβ* 1.001; 95% *CI* 0.540–1.463), SNSB language and related function scores (*aβ* 1.218; 95% *CI* 0.020–2.417), SNSB memory scores (*aβ* 1.963; 95% *CI* 0.841–3.084), SNSB frontal/executive function scores (*aβ* 1.715; 95% *CI* 0.401–3.029), amyloid PET SUVR (*aβ* −10.083; 95% *CI* −19.063 to −1.103), and MRI hippocampal volume (*aβ* 0.002; 95% *CI* 0.001–0.004).

**Table 3:** Association between stimulated oxygenation difference in the orbitofrontal cortex and cognitive impairment-related outcomes (secondary endpoints)

Variables	Oxygenation difference in the orbitofrontal cortex	Crude model		Adjusted model^a^	
		*β* (95% *CI*)	*P* value	*β* (95% *CI*)	*P* value
Mini-Mental State Examination	Olfactory stimulation	**0.957 (0.512 to 1.403)**	**<0.001**	**1.001 (0.540 to 1.463)**	**<0.001**
	None	−0.110 (−0.680 to 0.461)	0.704	0.007 (−0.569 to 0.583)	0.981
SNSB attention	Olfactory stimulation	1.654 (−0.426 to 3.734)	0.118	1.828 (−0.315 to 3.970)	0.179
	None	−0.618 (−3.098 to 1.861)	0.622	−0.440 (−2.916 to 2.037)	0.725
SNSB language and related function	Olfactory stimulation	**1.292 (0.129 to 2.454)**	**0.030**	**1.218 (0.020 to 2.417)**	**0.046**
	None	−0.795 (−2.192 to 0.601)	0.261	−0.961 (−2.367 to 0.445)	0.178
SNSB visuospatial function	Olfactory stimulation	0.679 (−0.076 to 1.434)	0.077	0.626 −0.155 to 1.408)	0.115
	None	−0.105 (−1.006 to 0.796)	0.818	−0.072 (−0.973 to 0.829)	0.875
SNSB memory	Olfactory stimulation	**1.830 (0.802 to 2.858)**	**0.001**	**1.963 (0.841 to 3.084)**	**0.001**
	None	−0.394 (−1.676 to 0.888)	0.544	−0.361 −1.717 to 0.994)	0.598
SNSB frontal/executive function	Olfactory stimulation	**1.817 (0.557 to 3.078)**	**0.005**	**1.715 (0.401 to 3.029)**	**0.011**
	None	−0.323 (−1.865 to 1.219)	0.678	−0.361 (−1.908 to 1.186)	0.644
Standard uptake value ratio	Olfactory stimulation	−**9.462 (**−**18.062 to** −**0.862)**	**0.031**	−**10.083 (**−**19.063 to** −**1.103)**	**0.028**
	None	0.391 (−9.878 to 10.660)	0.076	−2.514 (−12.817 to 7.790)	0.629
Hippocampal volume	Olfactory stimulation	**0.002 (0.001 to 0.003)**	**0.007**	**0.002 (0.001 to 0.004)**	**0.005**
	None	−0.001 (−0.003 to 0.001)	0.278	−0.001 (−0.002 to 0.001)	0.543

Numbers in bold indicate statistically significant associations (*P* < 0.05)

*Abbreviations*: *AD* Alzheimer’s disease, *CN* cognitively normal, *MCI* mild cognitive impairment, *SNSB* Seoul Neuropsychological Screening Battery

^a^Risk factors were adjusted for age, sex, education (continuous), household income (low, middle, and high), smoking (never or ex-smoker and current smoker), and Charlson comorbidity index (0, 1, and ≥2)

### Prediction model

**Fig. 2:**
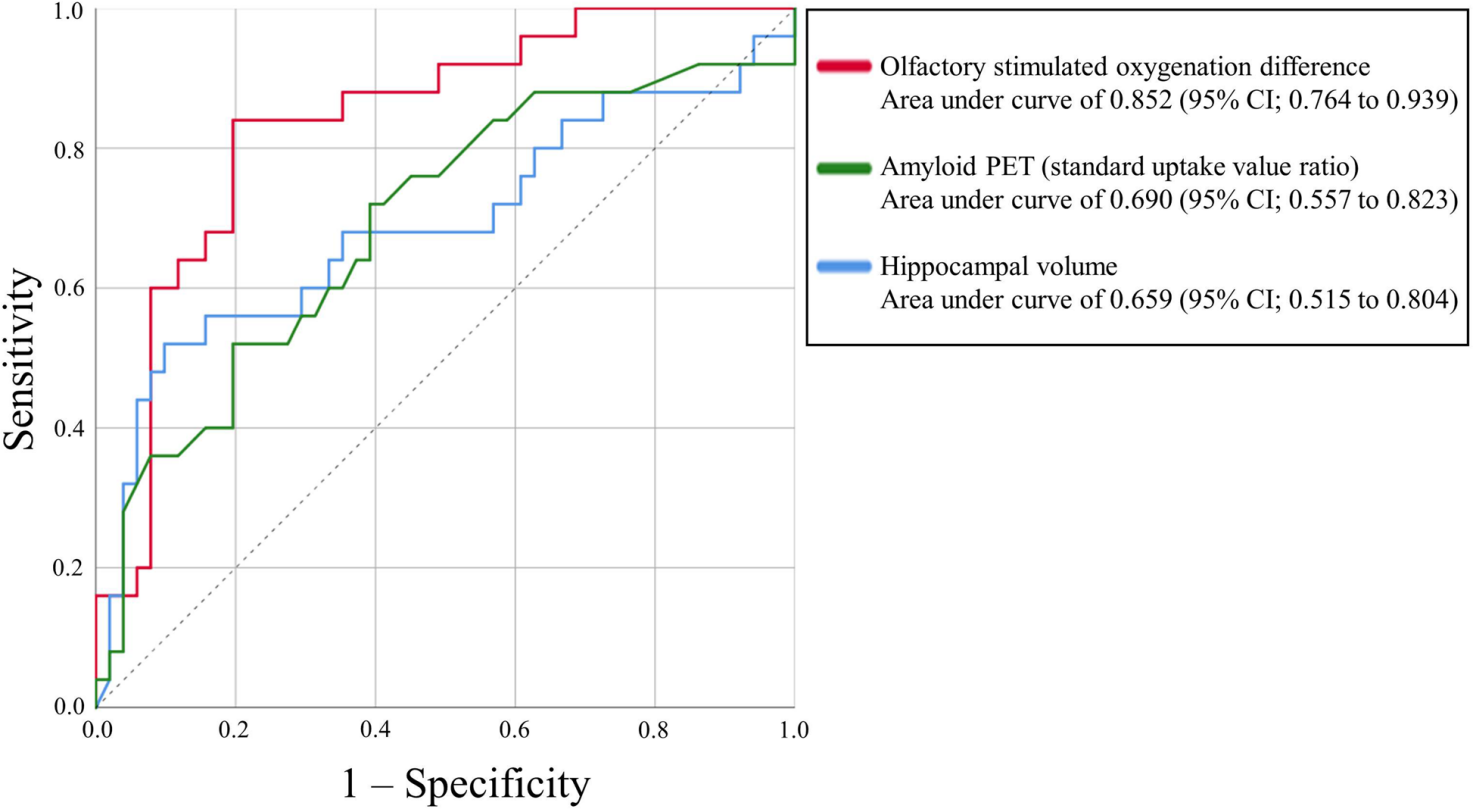
Receiver operating characteristic curves for various models of diagnosis of mild cognitive impairment in our cohort excluded patients with Alzheimer disease (*n* = 81)

The AUC for the diagnosis of AD was 0.837 (95% *CI* 0.753–0.921; sensitivity 100.0%; specificity 61.7%) using olfactory-stimulated oxygenation difference in the orbitofrontal cortex, 0.786 (95% *CI* 0.656–0.917; sensitivity 86.7%; specificity 81.3%) using amyloid PET SUVR, and 0.810 (95% *CI* 0.673–0.947; sensitivity 93.3%; specificity 57.1%) using MRI hippocampal volume (Table 4). The AUC for the diagnosis of both AD and MCI was 0.909 (95% *CI* 0.848–0.971; sensitivity 84.7%; specificity 94.4%) using olfactory-stimulated oxygenation difference in the orbitofrontal cortex, 0.793 (95% *CI* 0.694–0.893; sensitivity 89.8%; specificity 63.9%) using amyloid PET SUVR, and 0.758 (95% *CI* 0.644–0.871; sensitivity 98.1%; specificity 66.7%) using MRI hippocampal volume. Finally, the AUC for MCI diagnosis excluding patients with AD was the highest using olfactory-stimulated oxygenation difference in the orbitofrontal cortex (total *n* = 81; 0.903; 95% *CI* 0.836–0.970; sensitivity 95.2%; specificity 85.0%), compared with the amyloid PET SUVR (0.760; 95% *CI* 0.629–0.891; sensitivity 90.0%; specificity 56.1%) and MRI hippocampal volume (0.718; 95% *CI* 0.570–0.867; sensitivity 52.4%; specificity 91.5%) (Fig. 2). Finally, similar patterns of association were observed in the sensitivity analyses of differential conditions using alternative MCI definitions (Jak/Bondi typical criteria; Table S1 and S2) and another olfactory stimulation (leather-scented; Table S3).

**Table 4:** C-statistic for the prediction model in the diagnosis of AD or MCI^b^

	AUC	Sensitivity (%)	Specificity (%)
Prediction model as AD			
Olfactory-stimulated oxygenation difference in the orbitofrontal cortex	0.819 (0.736 to 0.902)	93.8	67.9
Standard uptake value ratio (amyloid PET)	0.786 (0.656 to 0.917)	93.3	57.1
Hippocampal volume (MRI)	0.810 (0.673 to 0.947)	86.7	81.3
Prediction model as AD and MCI			
Olfactory-stimulated oxygenation difference in the orbitofrontal cortex	0.873 (0.800 to 0.945)	88.1	81.8
Standard uptake value ratio (amyloid PET)	0.745 (0.640 to 0.850)	80.0	59.6
Hippocampal volume	0.733 (0.621 to 0.846)	61.0	92.6
Prediction model as MCI (excluded patients with AD)^a^			
Olfactory-stimulated oxygenation difference in the orbitofrontal cortex	0.852 (0.764 to 0.939)	84.6	81.8
Standard uptake value ratio (amyloid PET)	0.690 (0.557 to 0.823)	52.0	80.8
Hippocampal volume (MRI)	0.659 (0.515 to 0.804)	50.0	88.9

*Abbreviations*: *AD* Alzheimer’s disease, *AUC* area under the curve, *CN* cognitively normal, *MCI* mild cognitive impairment

^a^We excluded 16 patients with AD; therefore, the sample number for this analysis is 81

^b^The diagnostic criteria for MCI were based on the Jak/Bondi comprehensive criteria

## Discussion

### Findings of our study

Through a patient-level, single-group diagnostic trial, this is the first well-designed study for early detection of MCI and/or AD using novel fNIRS diagnostic techniques. We found that olfactory-stimulated oxygenation difference in the orbitofrontal cortex measured by fNIRS was associated with cognitive impairment, and its association was more pronounced with cognitive severity, whereas non-stimulated oxygenation difference was not associated with any cognitive impairment. In addition, the olfactory-stimulated oxygenation difference was associated with MMSE scores, SNSB language and related function scores, SNSB memory scores, SNSB frontal/executive function scores, SUVR from amyloid PET, and hippocampal volume from MRI. Finally, our results indicated that olfactory-stimulated oxygenation difference in the orbitofrontal cortex had diagnostic superiority for MCI and/or AD compared with amyloid PET or MRI scan.

### Comparison with previous studies

Previous diagnostic studies of MCI have used the fNIRS approach. In 2006, a preliminary study using the fNIRS approach during the verbal fluency task suggested the first potential diagnostic evidence for AD (total *n* = 47) [12]. Many researchers have provided several methods of stimulation using the fNIRS approach, including dual-task walking (*n* = 16) [13], resting state (*n* = 21) [14], N-back task (*n* = 24) [15], verbal fluency task (*n* = 61) [16], and memory and visuospatial test (*n* = 11) [17]. However, these previous studies have been conducted with small sample sizes and did not include comparisons with other diagnostic tests such as MRI, PET, or *APOE* genotypes, which may lead to difficulties in generating robust results and the interpretation of results. In addition, most of the stimulation methods used increased the burden of medical providers and subjects, unlike the olfactory stimulation method. Furthermore, our report is the first to reveal the diagnostic superiority of olfactory-stimulated fNIRS through direct comparison with other conventional imaging tests.

Furthermore, previous studies suggest that response patterns of olfactory stimulation are reflected in various modalities, such as fMRI [30] and EEG [31]. Changes in EEG patterns by olfactory stimulation are particularly characteristic in the FP1 and FP2 areas [31], which is similar to our results using the fNIRS approach in these areas.

### Possible explanations of our results

Previous studies have reported that olfactory dysfunction is an early symptom of cognitive impairment [32]. Previous post-mortem studies suggest that pathological changes in patients with AD involved pathological changes in the anterior olfactory nucleus and the olfactory bulb during the early stage of the disease, even before clinical symptoms manifest [33]. The Braak and Tredici hypothesis indicated that olfactory function might be vulnerable to AD and may affect disease progression [34]. Moreover, early neurodegenerative change patterns have been well established in AD according to their relationship with the olfactory bulb, prepiriform cortex, amygdala, entorhinal cortex, basal forebrain, raphe nuclei, locus coeruleus, and hippocampus [7]. Furthermore, previous epidemiologic studies suggest that olfactory function could predict neurodegeneration and decline in cognitive function through MRI [35] and PET [36] image findings and cognitive assessment questionnaires [37]. Therefore, many researchers have suggested that the olfactory function is a potential biomarker [7]. Furthermore, several epidemiologic studies have suggested that odor identification can predict the development of MCI [38]. Our study provides objective indicators for novel diagnostic tools along with the biological and epidemiological backgrounds linking olfactory function and AD. However, our study was preliminary with a small sample size and participants of only Asian ethnicity, and therefore, there are limitations of generalization and reproducibility.

### Policy implication

Our novel method can provide a non-invasive, high portability tool with lower cost, low radiation exposure, short examination time, fewer constraints on elderly patients who have difficulty sitting down for a long time or filling out questionnaires for examination, and excellent diagnostic superiority. A previous study suggested that the fNIRS approach can offer cognitive function assessment in resource-poor, rural communities [39], and our findings may reflect the value of this diagnostic tool that can be easily used not only in developed countries but also in underdeveloped countries.

### Strengths and limitations

Our study had some limitations. First, due to channel number restrictions to increase portability, the area of the fNIRS measurement was only the frontal cortex. Although our method provided excellent diagnostic results, olfactory-stimulated whole-head fNIRS measurement might be used to confirm the potential relationship and brain functional connectivity between other brain regions and AD [40]. Second, there are well-established ethnic differences in dementia risk [41] and our results are aimed at only Asians (Koreans); therefore, international research on ethnic differences in AD is needed for diagnostic validation. Third, although our study was an intervention trial, and for fNIRS to be used as a biomarker, we need a longitudinal study that includes repeated tests. Despite these limitations, our study has some strengths. Unlike previous studies, we enhanced the reproducibility and generalizability of our results through various imaging studies, *APOE* genotyping, and a larger sample size. In addition, we have provided diagnostic insights related to AD because we have conducted interventional trials from a medical perspective rather than from an engineering perspective. Finally, although we calculated the appropriate sample size for a diagnostic trial, we should be careful with the interpretation of the main results, which could be overestimated and skewed due to a small sample size. Therefore, future large-scale randomization diagnostic trials such as international multicenter trials are warranted.

## Conclusions

Through a patient-level, single-group, diagnostic intervention trial, this is the first well-designed study for the early detection of MCI and/or AD using novel olfactory-stimulated fNIRS diagnostic techniques. We found that olfactory-stimulated oxygenation difference in the orbitofrontal cortex had diagnostic superiority for MCI and/or AD, compared with amyloid PET or MRI scan. Our well-designed diagnostic trial suggests that the novel olfactory-stimulated fNIRS diagnostic technique may be a potential diagnostic tool for patients with MCI and/or AD and that large-scale randomized longitudinal trials of the diagnostic use of olfactory-stimulated fNIRS for AD are warranted.

## Supplementary Information

**Additional file 1:** Supplementary Methods and Results.

## Data Availability

The datasets used and analyzed during the current study are available from the corresponding author on reasonable request.
