# Supplementary material for: Novel diagnostic tools for identifying cognitive impairment using olfactory-stimulated functional near-infrared spectroscopy: patient-level, single-group, diagnostic trial
Source: Alzheimers Res Ther. 2022 Mar 8;14:39. doi: 10.1186/s13195-022-00978-w (PMC8905807; doi:10.1186/s13195-022-00978-w)
Supplement: Supplementary file 1 — Additional file 1: Supplementary Methods and Results. [file 13195_2022_978_MOESM1_ESM.docx]

**Supplementary Methods**

***Data acquisition***

The LED flickered at a rate of 10 Hz for each wavelength, and the PD had a sampling rate of 30 Hz (Figure S1). The light irradiated through the LED has three paths, as shown in Figure S2. Only a part of the path entered the diode. The light passes through the scalp and cortex, is absorbed by the two types of hemoglobin (oxidized hemoglobin and reduced hemoglobin) in the brain tissue, and directly enters the diode or is scattered without scattering in other tissues. If the signal (OD) entering the diode is processed using this method, the relative change in oxidized/reduced hemoglobin can be measured.

$\Delta$*OD_730_* $=\log\left( \frac{{LED}_{in}}{{LED}_{Out}} \right)\approx\varepsilon_{OxyHb}*\Delta C_{OxyHb}*d*DPF+\varepsilon_{DeoxyHb}*\Delta C_{DeoxyHb}*d*DPF$

$\Delta$*OD_850_* $=log(\frac{{LED}_{in}}{{LED}_{Out}})\approx\varepsilon_{OxyHb}*\Delta C_{OxyHb}*d*DPF+\varepsilon_{DeoxyHb}*\Delta C_{DeoxyHb}*d*DPF$

[OD: Amount of light attenuation measured by the detector according to wavelength, ε: extinction coefficient according to a specific wavelength, DPF: differential path length factor, C: concentration of hemoglobin, d: distance between LED and PD]

The value obtained by the above equation is a mixture of the skin and brain signals, as shown in Figures S3 and S4. Therefore, if the skin signal is removed from the channel that receives the overall signals, a pure brain signal can be obtained. The brain signal calculated in this way underwent wavelet transformation, and other physiological noises and noise caused by movement were removed with a low-pass filter (cut-off 0.4 Hz). Thereafter, the oxy-hemoglobin and deoxy-hemoglobin values calculated in this way were subtracted to obtain the oxygenation difference.

In the left and right channels, three oxygenation difference graphs were obtained for each channel, the olfactory stimulation value was the mean value of the olfactory stimulation section as the representative value, and none value was the mean value of the non-directional stimulation section as the representative value. The oxygenation difference value for each channel obtained in this way was examined for each group by finding the difference between values for the left and right three channels. The data processing procedure is summarized as follows (Figure S5).

***Experimental Paradigm***

All subjects underwent amyloid PET-CT and MRI within at least 1 year of fNIRS examination. In addition, SNSB and MMSE history listening tests were conducted. Investigators who performed this procedure conducted the test in an environment similar to that of a general clinic, as shown in Figure S6.

The olfactory stimulation was divided into unscented and scented stimulations. For odorless stimulation, an empty sniffstick pen was used. The protocol according to aroma stimulation is shown in Figure S7.

***Seoul Neuropsychological Screening Battery (SNSB)***

SNSB is a neuropsychological test developed in 2003 that measures attention, memory, language, spatiotemporal function, and frontal/executive function.[1] It is used to evaluate cognitive function in neurological diseases such as brain damage and dementia. SNSB is a Korean neurocognitive test method that has been researched and standardized in 447 older adults aged 55–80 years. SNSB includes clinical dementia grade, tests such as Barthel-activities, and Korea's instrumental daily life elderly depression scale. The detailed checklist of SNSB-II is as follows.[2-4]

| Cognitive Domain | SNSB-II |
| --- | --- |
| Attention | - Digit Span Test (forward and backward) - Letter cancellation - Vigilance test |
| Language & related functions | - Spontaneous speech, comprehension, and repetition - Korean-Boston Naming Test - Reading and writing - Finger naming and right-left orientation - Calculation and body-part identification |
| Visuospatial Functions | - Korean-Mini Mental State Examination (drawing) - Rey Complex Figure Test (copy) - Clock Drawing Test |
| Memory | - Korean-Mini Mental State Examination (registration and recall) - Seoul Verbal Learning Test-Elderly's version (immediate and delayed recalls, and recognition) - Rey Complex Figure Test (immediate and delayed recalls, and recognition) |
| Frontal/executive Functions | - Fist-edge-palm and alternating hand movement - Alternating square and triangle, and luria loop - Semantic (animal and supermarket), phonemic Controlled Oral Word Association Test - Korean-Color Word Stroop Test - Digit symbol Coding - Korean-Trail Making Test-Elderly version, - Korean-Color Word Stroop Test-60 |
| Other Indexes | - Korean-Mini Mental State Examination - Geriatric Depression Scale - Barthel-Activities of Daily Living - Clinical Dementia Rating - Global Deterioration Scale - Korean-Instrumental Activities of Daily Living |

The accuracy and diagnostic validity of SNSB have been proven through various studies.[2-4] For example, a previous study suggested that the sensitivity for the diagnosis of mild cognitive impairment of SNSB is 0.89 and specificity is 0.97 [5], and the sensitivity of diagnosis of mild cognitive impairment of Consortium to Establish a Registry for Alzheimer's Disease test is 79.2 and specificity is 71.0.[6]

***Korean Instrumental Activities of Daily Living (K-IADL)***

Functional evaluation of daily life is important when diagnosing dementia.[7] However, until 2002, there were few standardized measures of instrumental activity of daily living in Korea.[7] Therefore, the Korean Dementia Research Society created a Korean-style dementia daily life assessment with 11 questions.[7] In Korean Activities of Daily Living (K-IADL), these items were recombined into Korean items with reference to overseas IADL tools.[8-10]

This questionnaire consists of 11 items, which are scored on a scale of 0 to 3 points. 0 point means that you can do it alone, 1 point indicates requirement of some help, 2 points indicate requirement of a lot of help, and 3 points indicate an impossible task. Items include shopping, transportation use, financial management ability, housework management, food preparation, phone use, medication, recent memory, hobbies, watching TV, and housekeeping, adjusted by gender. K-IADL showed 83% sensitivity and 82% specificity in dementia patients [7-10]. The K-IADL is scored from 0 to 3 for each question and the detailed questions are as shown in the table below.

| K-IADL item | Corrected item-total  correlation |
| --- | --- |
| Shopping | 0.83 |
| Travel | 0.81 |
| Ability to handle finances | 0.86 |
| House keeping | 0.67 |
| Preparing food | 0.85 |
| Ability to use telephone | 0.78 |
| Responsibility for own medication | 0.81 |
| Recent Memory | 0.79 |
| Hobbies | 0.84 |
| Watching TV | 0.77 |
| Fixing around the house | 0.75 |

**Reference**

1. Kang Y, Na D, Hahn SJIHbr, co c: **Seoul neuropsychological screening battery**. 2003.

2. Ahn HJ, Chin J, Park A, Lee BH, Suh MK, Seo SW, Na DL: **Seoul Neuropsychological Screening Battery-dementia version (SNSB-D): a useful tool for assessing and monitoring cognitive impairments in dementia patients**. *J Korean Med Sci* 2010, **25**(7):1071-1076.

3. Lee JH, Lee KU, Lee DY, Kim KW, Jhoo JH, Kim JH, Lee KH, Kim SY, Han SH, Woo JI: **Development of the Korean version of the Consortium to Establish a Registry for Alzheimer's Disease Assessment Packet (CERAD-K): clinical and neuropsychological assessment batteries**. *The journals of gerontology Series B, Psychological sciences and social sciences* 2002, **57**(1):P47-53.

4. Joy S, Kaplan E, Fein D: **Speed and memory in the WAIS-III Digit Symbol--Coding subtest across the adult lifespan**. *Archives of clinical neuropsychology : the official journal of the National Academy of Neuropsychologists* 2004, **19**(6):759-767.

5. Kang IW, Beom IG, Cho JY, Son HR: **Accuracy of Korean-Mini-Mental Status Examination Based on Seoul Neuro-Psychological Screening Battery II Results**. *Korean journal of family medicine* 2016, **37**(3):177-181.

6. Seo EH, Lee DY, Lee JH, Choo IH, Kim JW, Kim SG, Park SY, Shin JH, Do YJ, Yoon JC *et al*: **Total scores of the CERAD neuropsychological assessment battery: validation for mild cognitive impairment and dementia patients with diverse etiologies**. *The American journal of geriatric psychiatry : official journal of the American Association for Geriatric Psychiatry* 2010, **18**(9):801-809.

7. Chin J, Park J, Yang SJ, Yeom J, Ahn Y, Baek MJ, Ryu HJ, Lee BH, Han NE, Ryu KH *et al*: **Re-standardization of the Korean-Instrumental Activities of Daily Living (K-IADL): Clinical Usefulness for Various Neurodegenerative Diseases**. *Dement Neurocogn Disord* 2018, **17**(1):11-22.

8. Lawton MP, Brody EM: **Assessment of older people: self-maintaining and instrumental activities of daily living**. *The Gerontologist* 1969, **9**(3):179-186.

9. Galasko D, Bennett D, Sano M, Ernesto C, Thomas R, Grundman M, Ferris S: **An inventory to assess activities of daily living for clinical trials in Alzheimer's disease. The Alzheimer's Disease Cooperative Study**. *Alzheimer disease and associated disorders* 1997, **11 Suppl 2**:S33-39.

10. Hindmarch I, Lehfeld H, de Jongh P, Erzigkeit H: **The Bayer Activities of Daily Living Scale (B-ADL)**. *Dementia and geriatric cognitive disorders* 1998, **9 Suppl 2**:20-26.


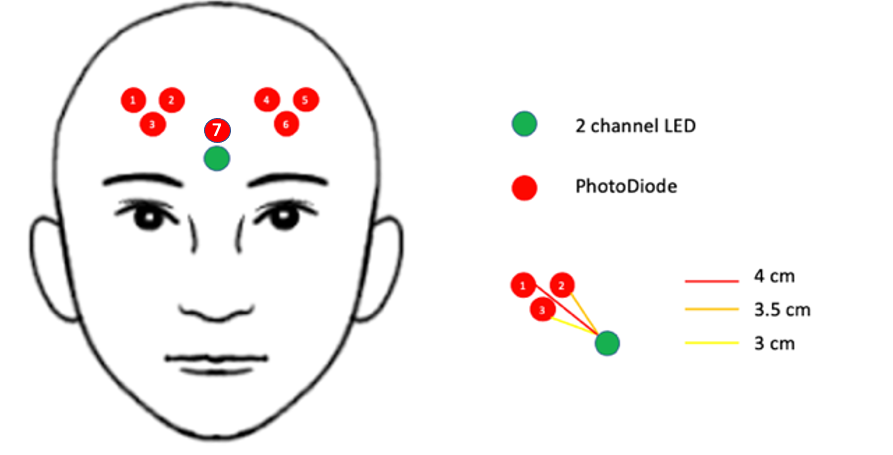
Figure S1. Schematic diagram of LED and PD attached to the patient during the experiment

Figure S2. The paths travelled by the light from the LED


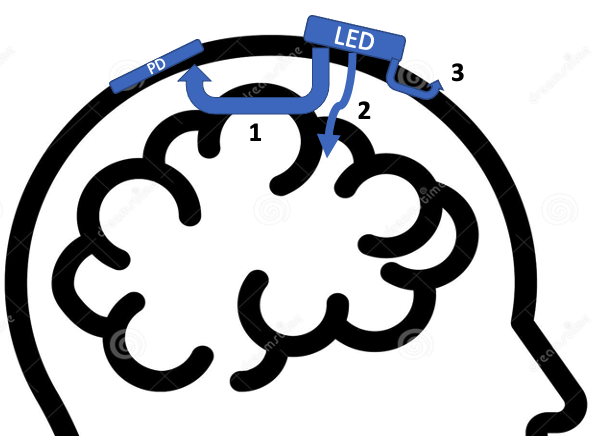
Pathway 1 is the path to the detector, and this path includes the fastest path and the scattering path. Pathway 2 is absorbed by hemoglobin in the tissue. Pathway 3 is the path reflected from the skin.

Figure S3. Algorithm to find brain signal; Corrected NIRS (C-NIRS)


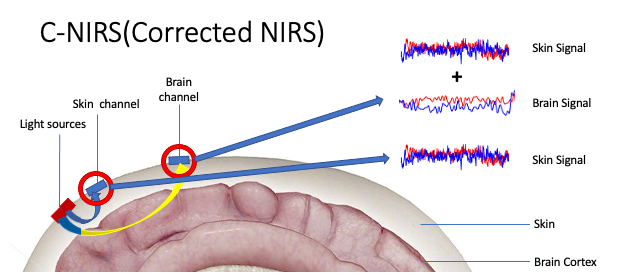


Figure S4. Oxygenation difference was calculated by subtracting deoxy-hemoglobin from oxy-hemoglobin.


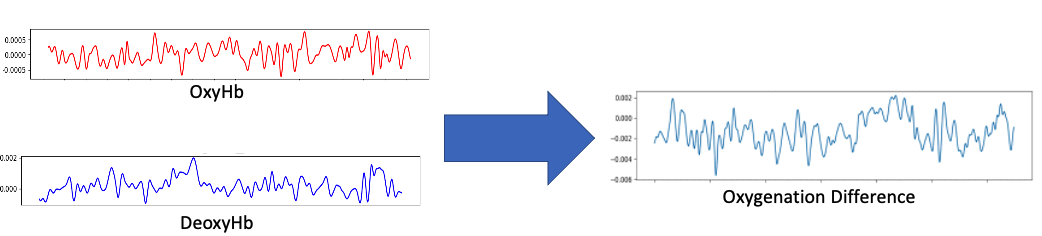


Figure S5. Data processing flow chart


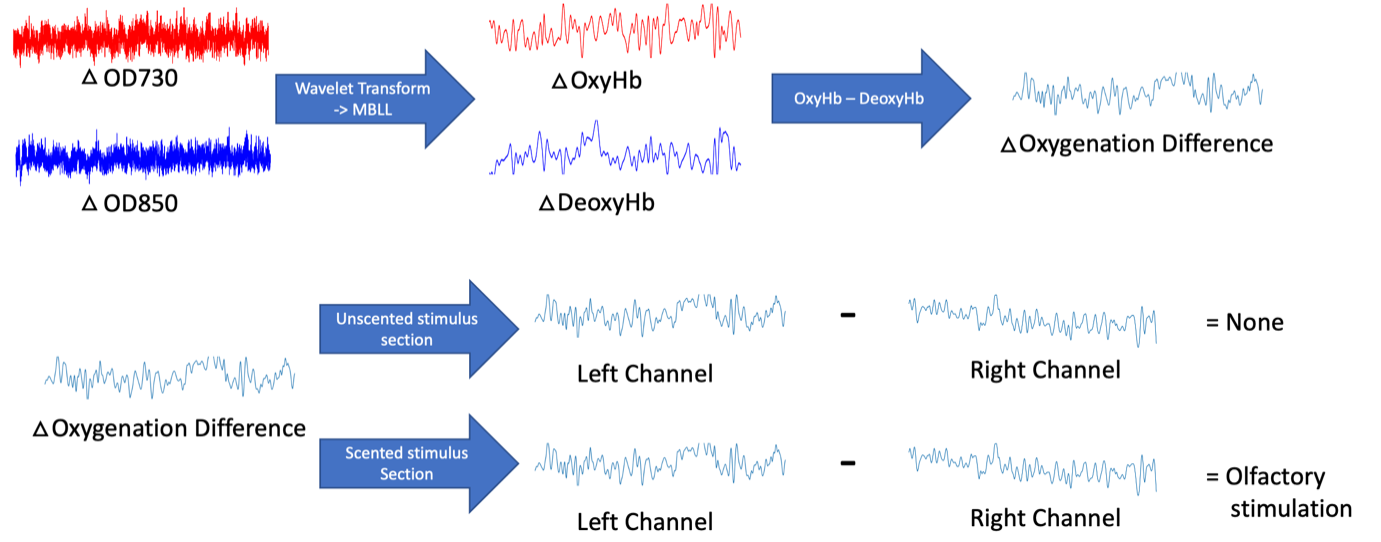


Figure S6. Subjects undergoing the experimental protocol


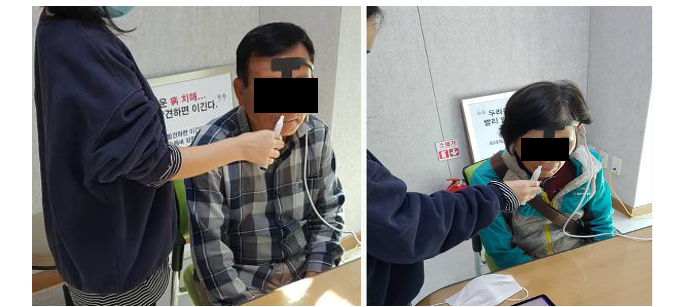


Figure S7. Olfactory stimulation method


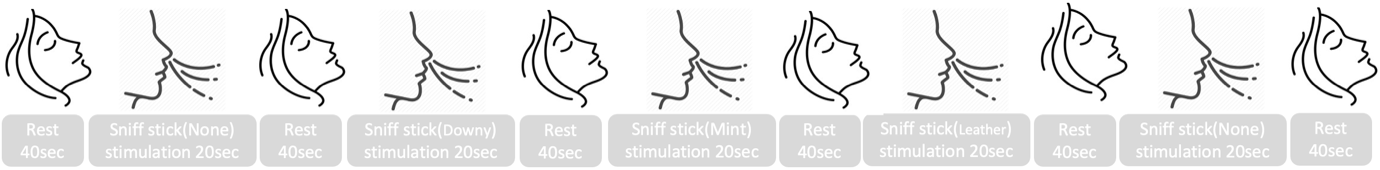


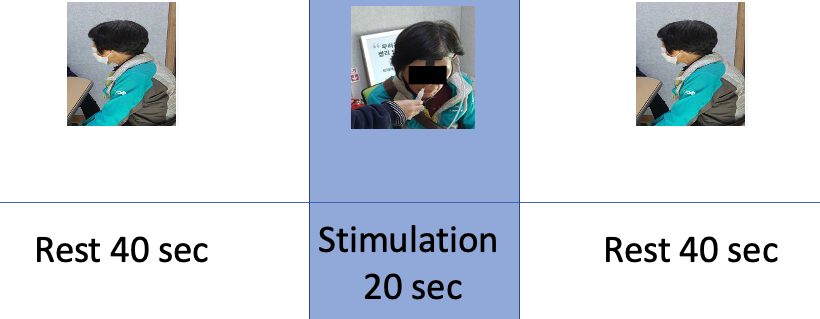


**Figure S8.** Instruction


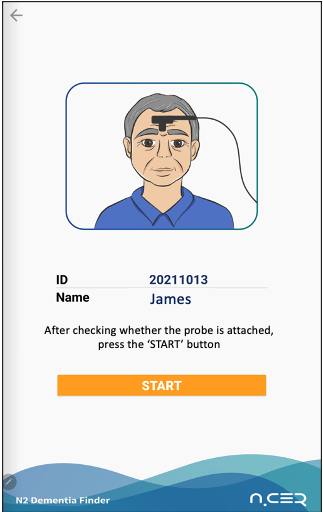


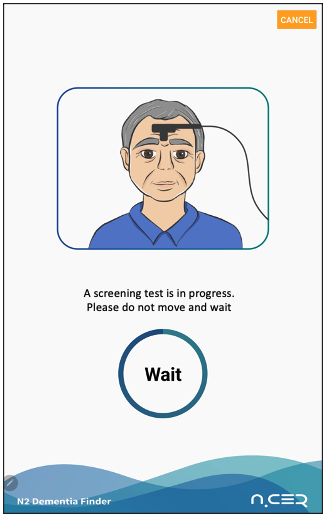


~~
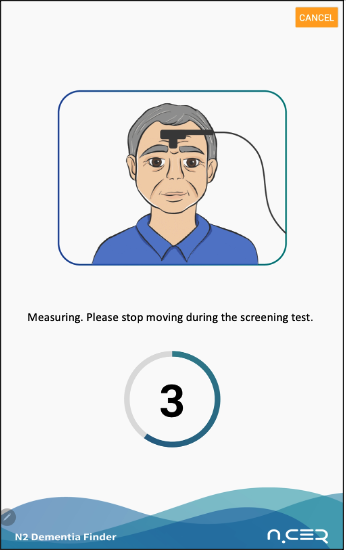
~~

~~
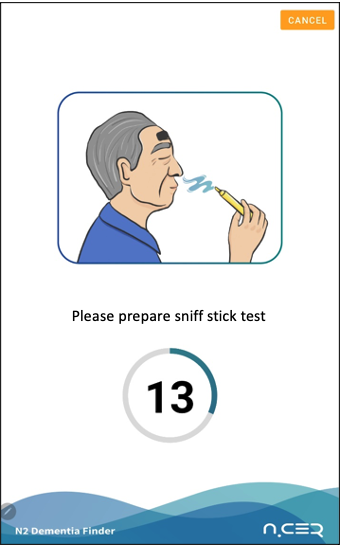
~~

~~
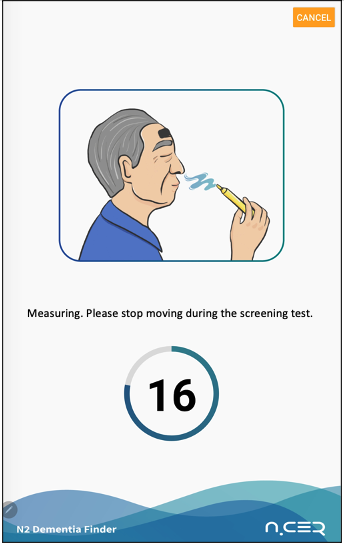
~~

Table S1. Association between stimulated oxygenation difference in the orbitofrontal cortex and cognitive impairment (primary endpoint) using alternative MCI definition^**^

| Oxygenation difference in orbitofrontal cortex | Model | CN (n=60) | MCI^**^ (n=21) | AD (n=16) |
| --- | --- | --- | --- | --- |
| Olfactory stimulation | Mean (95% CI) | 5.07 (2.31 to 7.83) | -2.79 (-3.69 to -1.89) | -4.21 (-5.82 to 2.60) |
|  | Adjusted Mean difference (model 1^*^) | 1.00 (reference) | **7.96 (3.62 to 12.30)** | **9.30 (4.46 to 14.13)** |
|  | Adjusted Mean difference (model 2^†^) | 1.00 (reference) | **8.73 (3.65 to 13.81)** | **10.79 (5.50 to 16.08)** |
| None | Mean (95% CI) | 1.01 (-2.07 to 4.09) | 3.75 (-1.34 to 8.84) | 0.93 (-1.71 to 3.57) |
|  | Adjusted Mean difference (model 1^*^) | 1.00 (reference) | -2.41 (-8.00 to 3.18) | 0.01 (-6.18 to 6.20) |
|  | Adjusted Mean difference (model 2^†^) | 1.00 (reference) | -2.37 (-7.85 to 3.11) | 0.72 (-5.39 to 6.82) |

Abbreviations: AD, Alzheimer disease; CN, cognitively normal; MCI, mild cognitive impairment.

^*^ Model 1 was adjusted for age and sex.

^†^ Model 2 was adjusted for age, sex, education (continuous), household income (low, middle, and high), smoking (never or ex-smoker and current smoker), Charlson comorbidity index (0, 1, and ≥2).

^**^ The diagnostic criteria for MCI were based on the Jak/Bondi typical criteria.

Numbers in bold indicate statistically significant associations (*P* < 0.05).

Table S2. C-statistic for the prediction model in the diagnosis of AD or MCI^†^ using alternative MCI definition.

|  | AUC | Sensitivity (%) | Specificity (%) |
| --- | --- | --- | --- |
| Prediction model as AD |  |  |  |
| Olfactory stimulated oxygenation difference in the orbitofrontal cortex | 0.837 (0.753 to 0.921) | 100.0 | 61.7 |
| Standard uptake value ratio (amyloid PET) | 0.786 (0.656 to 0.917) | 86.7 | 81.3 |
| Hippocampal volume (MRI) | 0.810 (0.673 to 0.947) | 93.3 | 57.1 |
| Prediction model as AD and MCI |  |  |  |
| Olfactory stimulated oxygenation difference in the orbitofrontal cortex | 0.909 (0.848 to 0.971) | 84.7 | 94.4 |
| Standard uptake value ratio (amyloid PET) | 0.793 (0.694 to 0.893) | 89.8 | 63.9 |
| Hippocampal volume | 0.758 (0.644 to 0.871) | 98.1 | 66.7 |
| Prediction model as MCI (excluded patients with AD)* |  |  |  |
| Olfactory stimulated oxygenation difference in the orbitofrontal cortex | 0.903 (0.836 to 0.970) | 95.2 | 85.0 |
| Standard uptake value ratio (amyloid PET) | 0.760 (0.629 to 0.891) | 90.0 | 56.1 |
| Hippocampal volume (MRI) | 0.683 (0.529 to 0.838) | 52.4 | 91.5 |

Abbreviations: AD, Alzheimer disease; AUC, Area under the curve; CN, cognitively normal; MCI, mild cognitive impairment.

^*^ We excluded 16 patients with AD, therefore, the sample number for this analysis is 81.

^†^ The diagnostic criteria for MCI were based on the Jak/Bondi typical criteria.

Table S3. Association between stimulated oxygenation difference in the orbitofrontal cortex and cognitive impairment (primary endpoint)

| Oxygenation difference in orbitofrontal cortex | Model | CN | MCI^**^ | AD | P*trend* |
| --- | --- | --- | --- | --- | --- |
| Olfactory stimulation (peppermint-scented) | Mean (95% CI) | 5.94 (3.13 to 8.74) | -0.22 (-2.48 to 2.04) | -3.96 (-5.69 to -2.23) |  |
|  | Adjusted Mean difference (model 1^*^) | 1.00 (reference) | **6.12 (2.12 to 10.13)** | **9.93 (5.10 to 14.76)** | **<0.001** |
|  | Adjusted Mean difference (model 2^†^) | 1.00 (reference) | **6.83 (3.15 to 10.51)** | **9.92 (5.72 to 14.13)** | **<0.001** |
| Olfactory stimulation (leather-scented) | Mean (95% CI) | 5.94 (3.71 to 8.16) | -3.11 (-6.34 to 0.12) | -4.21 (-8.33 to -0.09) |  |
|  | Adjusted Mean difference (model 1^*^) | 1.00 (reference) | **9.00 (5.10 to 12.91)** | **10.17 (5.49 to 14.86)** | **<0.001** |
|  | Adjusted Mean difference (model 2^†^) | 1.00 (reference) | **8.99 (5.08 to 12.92)** | **9.96 (5.24 to 14.69)** | **<0.001** |
| None | Mean (95% CI) | 1.63 (-1.67 to 4.93) | 1.92 (-2.46 to 6.30) | 0.93 (-1.71 to 3.57) |  |
|  | Adjusted Mean difference (model 1^*^) | 1.00 (reference) | -0.47 (-5.57 to 4.63) | 1.32 (-4.83 to 7.46) | **0.872** |
|  | Adjusted Mean difference (model 2^†^) | 1.00 (reference) | -0.39 (-7.05 to 6.26) | 0.51 (-7.11 to 8.12) | **0.870** |

Abbreviations: AD, Alzheimer disease; CN, cognitively normal; MCI, mild cognitive impairment.

^*^ Model 1 was adjusted for age and sex.

^†^ Model 2 was adjusted for age, sex, education (continuous), household income (low, middle, and high), smoking (never or ex-smoker and current smoker), Charlson comorbidity index (0, 1, and ≥2).

^**^ The diagnostic criteria for MCI were based on the Jak/Bondi comprehensive criteria.

Numbers in bold indicate statistically significant associations (*P* < 0.05).
